# The preventive surgical site infection bundle in patients with colorectal perforation

**DOI:** 10.1186/s12893-015-0115-0

**Published:** 2015-12-18

**Authors:** Takehito Yamamoto, Takeshi Morimoto, Ryosuke Kita, Hideyuki Masui, Hiromitsu Kinoshita, Yusuke Sakamoto, Kazuyuki Okada, Junji Komori, Akira Miki, Masato Kondo, Kenji Uryuhara, Hiroyuki Kobayashi, Hiroki Hashida, Satoshi Kaihara, Ryo Hosotani

**Affiliations:** Department of Surgery, Kobe City Medical Center General Hospital, 2-1-1, Minatojima-Minamimachi, Chuo-ku Kobe, Japan; Department of Gastroenterological Surgery and Oncology, Kitano Hospital, The Tazuke Kofukai Medical Research Institute, 2-4-20 Ogimachi, Kita-ku, Osaka, Japan; Department of Clinical Epidemiology, Hyogo College of Medicine, 1 Mukogawa, Nishinomiya, Hyogo Japan; Clinical Research Center, Kobe City Medical Center General Hospital, 2-1-1 Minatojima-Minamimachi, Chuo-ku Kobe, Japan

**Keywords:** Abdominal infection, Surgical site infection, Wound infection, Perforation, Peritonitis

## Abstract

**Background:**

Incisional surgical site infection (SSI) is one of the most frequent complications that occur after colorectal surgery. Surgery for colorectal perforation carries an especially high risk of incisional SSI because fecal ascites contaminates the incision intraoperatively, and in patients who underwent stoma creation, the incision is located near the infective origin and is subject to infection postoperatively. Although effectiveness of the preventive SSI bundle of elective colorectal surgery has been reported, no study has focused exclusively on emergency surgery for colorectal perforation.

**Methods:**

Patients with colorectal perforation who underwent emergency surgery and stoma creation from 2010 to 2015 at our center were consecutively enrolled in the study. In March 2013, we developed the preventive incisional SSI bundle for patients with colorectal perforation undergoing stoma creation. The effectiveness of the bundle in these patients was determined and the rates of incisional SSI between before and after March 2013 were compared.

**Results:**

We enrolled 108 patients with colorectal perforation who underwent emergency operation during the study period. Thirteen patients were excluded because they died within 30 days after surgery, and 23 patients without stoma were excluded; thus, 72 patients were analyzed. There were 47 patients in the pre-implementation group and 25 patients in the post-implementation group. The rate of incisional SSI was significantly lower after implementation of preventive incisional SSI bundle (43 % vs. 20 %, *p* = 0.049). Postoperative hospital stay was significantly shorter after implementation of the bundle (27 vs. 18 days respectively; *p* = 0.008).

**Conclusions:**

The preventive incisional SSI bundle was effective in preventing incisional SSI in patients with colorectal perforation undergoing emergency surgery with stoma creation.

## Background

Surgical site infection (SSI) is one of the most common hospital-acquired complications in surgical patients. Colorectal surgery has been reported to have an SSI rate of 9–41 % [1–5], which is higher than that of gastrointestinal surgery. Moreover, SSI is significantly associated with longer hospital stay, which in turn results in higher inpatient costs [6–9]. Mahmoud et al. retrospectively analyzed 25,825 patients and reported that SSI was significantly and independently associated with longer hospital stay and increased costs [8]. Determining the strategies for its prevention could therefore improve patient care while lowering the duration and cost of hospital stay in patients at risk.

The widespread dissemination of bacteria throughout the intra-abdominal space that results from colorectal perforation often leads to panperitonitis. Surgical treatment of colorectal perforation can expose the ascites to fecal contamination, which in turn contaminates the incision. Moreover, in patients undergoing stoma creation, the incisional wound is located near the infective origin and is subject to infection postoperatively. Thus, patients with colorectal perforation undergoing stoma creation are at extremely high risk of incisional SSI, and both the intraoperative and postoperative wound management should be carefully done. Although the rate of incisional SSI is reportedly higher in emergency than in elective colorectal surgery [10], no report has focused exclusively on patients who underwent emergency surgery with stoma creation for colorectal perforation. In March 2013, we implemented a preventive incisional SSI bundle for wound management of this patient population. We sought to evaluate our preventive SSI bundle in patients undergoing emergency surgery with stoma creation for colorectal perforation.

## Methods

### Patient

Among patients with colorectal perforation who underwent emergency surgery at a single center from 2010 to 2015, those who underwent stoma creation were enrolled consecutively in this study. Patients with a perforated appendix related to appendicitis were excluded. Patients who died following surgery were excluded. The primary outcome was occurrence of postoperative incisional SSI.

The following patient data were collected: age, sex, body mass index, comorbidities, preoperative vital signs (body temperature, systolic blood pressure, and heart rate), preoperative laboratory values (white blood cell [WBC] count, C-reactive protein [CRP], and serum albumin and creatinine), etiology and site of the perforation, duration of surgery (min), and volume of intraoperative blood loss (mL).

The study protocol was approved by the Institutional Review Board of Kobe City Medical Center General Hospital. Informed consent was waived because this study was conducted retrospectively.

### Diagnosis

Colorectal perforation was diagnosed by two or more surgeons and radiologists according to the following criteria: a) the presence of symptoms indicating panperitonitis, such as severe abdominal pain and nausea; b) the presence of peritoneal irritation signs indicating panperitonitis; for example, abdominal guarding and rebound tenderness; and c) the presence of free air on preoperative computed tomography. The findings of abdominal radiography were not useful in diagnosing colorectal perforation and were therefore not included in the diagnostic criteria. All perforations were diagnosed preoperatively and confirmed intraoperatively.

The surgical wound was examined by a physician and a nurse at least once a day and by the SSI surveillance team of our institution once a week until discharge of the patient from the hospital. After discharge, the wound was examined by an outpatient doctor and a nurse until 30 days after surgery. The diagnosis of SSI was made after discussions with surgeons, nurses, and members of the SSI surveillance team and was based on the definition included in the guidelines of the Centers for Disease Control and Prevention [11].

### Preventive incisional SSI bundle

**Fig. 1:**
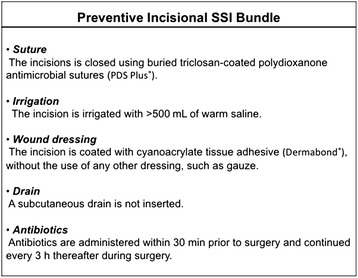
Preventive Incisional surgical site infection (SSI) bundle

In March 2013, we implemented a preventive incisional SSI bundle for wound management of patients undergoing stoma creation. The bundle consisted of the following steps (Fig. 1): a) The incision is closed using buried triclosan-coated polydioxanone antim median [range] icrobial sutures (PDS® Plus Antibacterial Suture; Ethicon, Inc., Somerville, NJ, USA); b) the incision is irrigated with > 500 mL of warm normal saline solution; c) the incision is coated with cyanoacrylate tissue adhesive (Dermabond®; Ethicon, Inc.) without the use of any other dressing, such as gauze; d) a subcutaneous drain is not inserted; and e) antibiotics are administered within 30 min prior to surgery and are readministered every 3 h during surgery.

Before implementation of the bundle, the kinds of sutures and dressings, and whether or not a subcutaneous drain was inserted were dependent on each operator. The volume of normal saline solution and the timing of antibiotics administration were not consistent. After March 2013, all cases included conformed to this bundle.

Thus, we compared the rates of incisional SSI before and after March 2013, which allowed us to analyze the effectiveness of the bundle.

### Statistical analyses

Continuous variables are presented as mean ± standard deviation or median [range], and categorical variables as number and percentage. To compare the two groups, the *χ*^2^ and Mann–Whitney U tests were used. All statistical analyses were conducted by one of the physicians participating in the study (TY), under the supervision of the chief statistician of our institution (TM), using JMP®, Version 10 (SAS Institute Inc., Cary, NC, USA). All reported *p* values are two-sided. A *p* value < 0.05 was considered statistically significant.

## Results

**Fig. 2:**
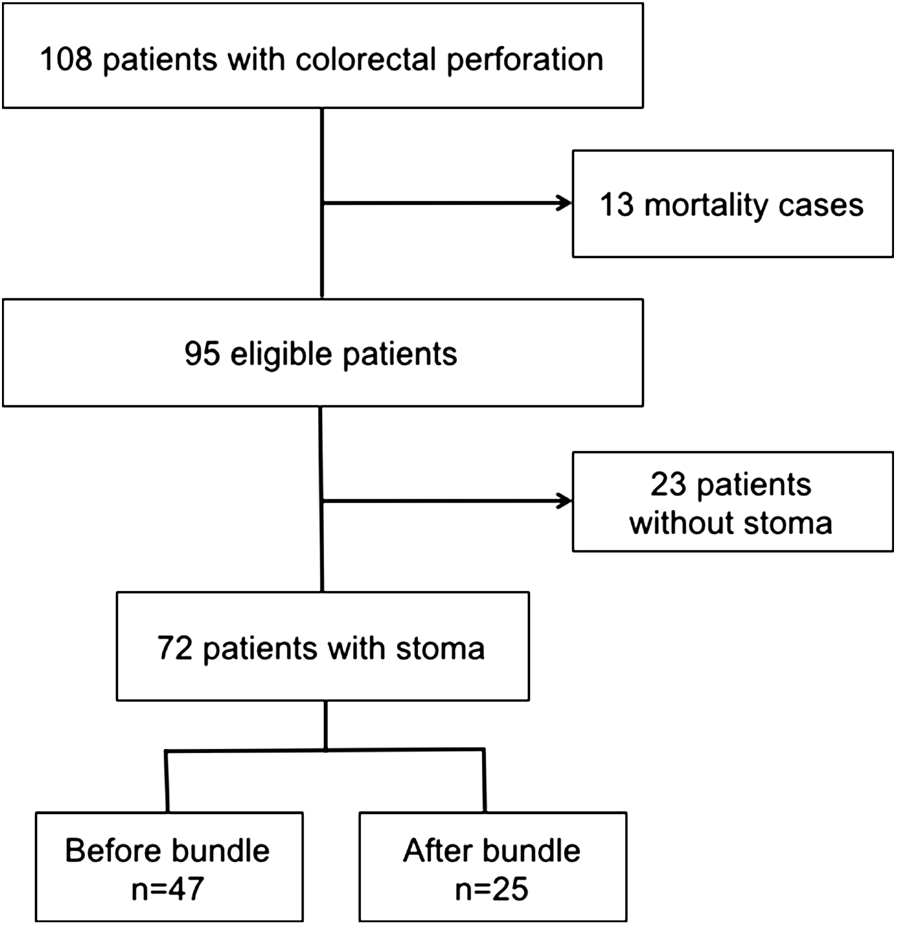
Patient selection

We enrolled 108 consecutive patients with colorectal perforation who underwent emergency operation during the study period. Thirteen patients were excluded because they died within 30 days after surgery, and 23 patients without stoma were excluded; thus, 72 patients were analyzed. There were 47 patients in the pre-implementation group and 25 patients in the post-implementation group. Patient selection is shown in Fig. 2.

Comparison of clinical characteristics between the pre-implementation group and the post-implementation group is presented in Table 1. There was no statistical difference in the two groups. Comparison of the rates of incisional SSI and postoperative hospital stay between the pre-implementation group and the post-implementation group is shown in Table 2. Patients treated after implementing the bundle had a significantly lower rate of incisional SSI than did those treated before implementation of the bundle (43 % vs. 20 %, respectively; *p* = 0.049). Postoperative hospital stay was significantly shorter after implementation of the bundle (27 vs. 18 days respectively; *p* = 0.008).

**Table 1:** Comparison of the characteristics of patients before and after implementation of the preventive incisional SSI bundle

Variable	Before (*n* = 47)	After (*n* = 25)	*p* value
Age (years)	73 ± 12	69 ± 12	0.216
Males	19 (40.4)	18 (72.0)	0.051
Body mass index (kg/m^2^)	21.1 ± 4.1	21.6 ± 4.6	0.508
Chemotherapy	3 (6.4)	3 (12.0)	0.412
Steroid use	11 (23.4)	8 (32.0)	0.431
Diabetes mellitus	3 (6.4)	1 (4.0)	0.660
Body temperature > 38.0 °C or < 36.0 °C	29 (61.7)	21 (84.0)	0.051
Systolic blood pressure (mmHg)	127 ± 29	127 ± 27	0.723
Heart rate (beats per min)	99 ± 19	97 ± 18	0.838
WBC count >12,000/μL or < 4,000/μL	23 (48.9)	9 (36.0)	0.293
CRP (mg/dL)	12.6 ± 10.5	13.2 ± 12.4	0.995
Alb (mg/dL)	2.7 ± 0.6	2.8 ± 0.9	0.850
Cr (mg/dL)	1.3 ± 1.6	1.1 ± 0.8	0.696
Cause of perforation			0.291
Diverticulum	15 (31.9)	10 (40.0)	
Cancer	10 (21.3)	3 (12.0)	
Fecal impaction	7 (14.9)	5 (20.0)	
Iatrogenic	5 (10.6)	0	
Inflammatory disease	1 (2.1)	3 (12.0)	
Trauma	1 (2.1)	1 (8.0)	
Idiopathic	8 (17.0)	3 (12.0)	
Perforation site			0.454
Cecum	2 (4.2)	0	
Ascending colon	0	0	
Transverse colon	4 (8.5)	0	
Descending colon	2 (4.2)	1 (8.0)	
Sigmoid colon	31 (64.6)	20 (80.0)	
Rectum	2 (4.3)	4 (16.0)	
Duration of surgery (min)	181 ± 57	193 ± 48	0.394
Blood loss (mL)	229 [0–3112]	196 [0–1231]	0.749

Data presented as mean ± standard deviation, median [range], or n (%). *Alb* albumin, *BMI* body mass index, *Cr* creatinine, *CRP* C-reactive protein, *SD, SSI* surgical site infection, *WBC* white blood cell

**Table 2:** Comparison of the rates of incisional SSI and postoperative hospital stay between before and after implementation of the preventive incisional SSI bundle

Variable	Before (*n* = 47)	After (*n* = 25)	*p* value
Incisional SSI	20 (42.6)	5 (20.0)	0.049*
Postoperative hospital stay (day)	27 [11–185]	18 [4–150]	0.008*

*p < 0.05

Data presented as n (%) or median [range]

*SSI* surgical site infection

## Discussion

A number of previous studies identified stoma creation to be one of the important risk factors for incisional SSI after colorectal surgery [1, 12, 13]. For example, in their analysis of 217 patients who underwent rectal surgery, Konishi et al. identified stoma creation as a risk factor for incisional SSI (OR, 4.9) [1]. However, those studies included many kinds of colorectal diseases; bowel obstruction caused by cancer, volvulus, and invagination; appendicitis; Crohn’s disease; and so on. Because of the differences in the risks of SSI among colorectal diseases and different levels of preventive measures required for each, expensive tools such as antimicrobial sutures and cyanoacrylate tissue adhesive would not be necessary for diseases with less risk of incisional SSI. Therefore, we focused on the patient population with the highest risk of incisional SSI; that is, the patients who underwent emergency surgery with stoma creation for colorectal perforation.

The effectiveness of preventive SSI bundle in colorectal surgery has been proved in many studies [14–17]. Tanner et al. performed a systematic review and meta-analysis including 8515 patients in 13 studies, and they reported that the surgical care bundles significantly reduced the risk of SSI in patients undergoing colorectal surgery [16]. However, no study focused exclusively on emergency surgery for colorectal perforation. We considered that preventive incisional SSI bundle was needed especially for the patients with high risk of incisional SSI. Therefore, we implemented the bundle for this patient population in March 2013, and proved in the present study that it was also effective for the patients who underwent emergency surgery with stoma creation for colorectal perforation.

In patients with stoma, the incision site is near the infective origin, which can lead to fecal contamination of the wound. Therefore, we reasoned that cyanoacrylate tissue adhesive (Dermabond®; Ethicon, Inc.) used as a wound dressing during stoma creation would provide an effective barrier against microorganism penetration. We therefore consider the use of cyanoacrylate tissue adhesive as a microbial barrier to have played a major role in our bundle for preventing incisional SSI.

Although this approach has not been analyzed sufficiently in clinical studies, its in vitro effectiveness has been reported by Bhenede et al. [18], who used an agar medium containing a dye that changed color in the presence of acidic microbial metabolic products. The authors applied a uniformly thick film of cyanoacrylate adhesive on the surface of the agar and found that it served as a barrier that prevented microbial contamination. This result suggested the clinical benefit of using a cyanoacrylate adhesive in the prevention of incisional SSI after colorectal surgery.

The effectiveness of triclosan-coated sutures, which are used in our preventive incisional SSI bundle, has been demonstrated in two meta-analyses; one by Apisarnthanarak et al. [19], which consisted of 22 randomized controlled trials (RCTs) and seven non-RCTs, and one by Wang et al. [20], which included 17 RCTs. Both teams found that the use of triclosan-coated sutures significantly reduced the rate of incisional SSI. The studies analyzed included patients undergoing breast surgery, cardiac surgery, and emergency surgery for a contaminated abdomen, but the effectiveness of triclosan-coated sutures in patients undergoing surgery for colorectal perforation was not determined. At our institution, in operations other than those involving stoma creation, the types of sutures used in wound closure vary depending on the surgeon. Therefore, the broader benefit of triclosan-coated sutures remains to be confirmed.

The bundle comprising intraoperative wound irrigation, subcutaneous drain, and antibiotics is not specific to surgery for colorectal perforation but is common to other gastroenterological surgeries in our institution. There is conflicting evidence regarding the association between subcutaneous drain insertion and incisional SSI. Fujii et al. analyzed 79 obese patients who underwent colorectal surgery and recommended the use of a subcutaneous drain [21]. Conversely, Baier et al. reported in their RCT of 200 patients that a subcutaneous drain did not reduce the rate of incisional SSI [22]. Similarly, in an RCT comparing patients with (*n* = 210) and without drains (*n* = 192), Kaya et al. found that the use of subcutaneous drain was not effective in preventing incisional SSI, but their subgroup analyses of colorectal malignancies and lower abdominal incisions indicated the usefulness of a subcutaneous drain [23]. Therefore, the use of subcutaneous drains in surgery for colorectal perforation remains controversial, and further studies should be performed.

There are few reports about the techniques of subcutaneous tissue irrigation. Cervantes-Sánchez et al. reported that 300 ml of saline solution using a 20-ml syringe significantly reduced the SSI rate after surgery for appendicitis, while Horiuchi et al. analyzed 272 patients who underwent gastrointestinal surgery and 500–1,000 mL of saline was used to irrigate the subcutaneous tissue in their study [24, 25]. Standard protocol for dirty operations by Watanabe et al. in their analysis about the emergency colorectal surgery adopted irrigation of subcutaneous tissue with 500 mL of warm saline [3]. Referring to these reports, we considered that irrigation with warm saline >500 ml was enough in the bundle for colorectal perforation.

The use of prophylactic antibiotics in the bundle conformed to the guideline of Centers for Disease Control and Prevention (CDC) [11]. This was the same protocol as other similar studies about SSI after colorctal surgery [1, 3].

Our study had several limitations. First, operative and postoperative management was performed by different clinicians, resulting in an inconsistent quality of care. Second, the study was conducted at a single center, and the number of patients was small. A large-scale, multicenter study will be needed to confirm our findings.

## Conclusion

The preventive incisional SSI bundle was effective in preventing incisional SSI in patients with colorectal perforation who underwent stoma creation.
